# Book Review: Reflexive Pronouns: A Theoretical and Experimental Synthesis

**DOI:** 10.3389/fpsyg.2021.716742

**Published:** 2021-07-08

**Authors:** Li Zeng, Hao Lin, Xinmin Zheng

**Affiliations:** ^1^School of Education, Shanghai International Studies University, Shanghai, China; ^2^Institute of Linguistics, Shanghai International Studies University, Shanghai, China

**Keywords:** reflexive pronouns, Emergentive Reflexive Approach, binding, experiment, RNGPTA, pragmatics

Authored by Darcy Sperlich, *Reflexive Pronouns: A Theoretical and Experimental Synthesis* is a book in which the author proposes an innovative approach, i.e., the Emergentive Reflexive Approach (ERA) model, to capture the complexity of reflexive interpretation cross languages.

The book consists of four chapters. Chapter One briefly introduces the ERA model and lays out the research scope of the reflexives in six languages, namely, English, Dutch, German, Chinese, Japanese, and Korean. Next, it carefully defines the key terminologies and morphological features of these reflexives as well as the sentence structures involved. Chapter Two, “A Theoretical Synthesis,” offers a detailed survey of the reflexive data of the six languages in the theoretical literature. Then, it compares and critiques two recent generative proposals of reflexive interpretation with their conceptual issues and limitations on data coverage. After that, it proposes the ERA model, which is constructed on O'Grady's (2005) sentence processor and Huang's (2000) RNGPTA (Revised Neo-Gricean Pragmatic Theory of Anaphora) pragmatic processor. The ERA model's application to account for the main reflexive interpretation patterns in the six languages is also reported. Chapter Three, “An Experimental Synthesis,” synthesizes the key empirical findings drawn from first and second language acquisition, bilingual, psycholinguistic, neurolinguistic, and clinical studies. With a thorough analysis of these findings, the author observed distinct differences between English, Dutch, and German reflexive pronouns on the one hand and Chinese, Japanese, and Korean reflexive pronouns on the other. The former are generally processed via the sentence processor, whereas the latter are dominantly subject to pragmatic processing. The different processing patterns, according to the author, lend empirical support to his grouping of the three syntactic languages (i.e., English, Dutch and German) and that of the three pragmatic languages (i.e., Chinese, Japanese, and Korean) with respect to reflexive interpretation. Chapter Four, “A Final Synthesis,” elaborates how the ERA model can be applied to researching the six languages mentioned above and beyond. At the end of the chapter, further research directions on reflexive pronouns are suggested.

First of all, as a forceful attempt to extend Huang's (2000) RNGPTA, the book presents a meticulous examination of crosslinguistic data with a rigorous methodology. It surveys languages of different typological types and draws on findings from empirical studies concerning reflexive interpretation. These lend significant credits to the ERA model's descriptive adequacy. On top of this, the author follows Huang (2016) to conceptualize natural languages as two distinct classes concerning reflexive interpretation. It is obvious that this original and holistic view can shed new light on the mechanism of reflexive interpretation in a variety of languages.

Besides, the theoretical significance of the ERA model also earns our attention. We understand that the model discards the conventional theory-internal (i.e., reflexive-specific) rules in exchange for derived principles from the neo-Gricean theory of generalized conversational implicature to explain reflexive interpretation. Apparently, it is theoretically more desirable and cognitively more economical. In our opinion, the model would have been more persuasive and useful, though, if the author had delineated the division line between the two sets of languages (i.e., syntactic vs. pragmatic one) in greater detail. Moreover, the ERA model goes with the current theoretical tide to incorporate both pragmatic and syntactic factors to account for reflexive interpretation, strengthening its accountability for more nuanced aspects of reflexives, which we think is also an undeniable contribution of this book.

Furthermore, the book goes beyond the theoretical discussion and explores the applicability of the ERA model to the empirical realm on first and second language acquisition, neurolinguistics, and clinical studies. This accords with the current trend that the linguistic theory can accommodate the psychological reality of language. Indeed, the ERA's unified solution to the complexity of reflexive interpretation is also more helpful and more inclusive to inform empirical studies in bilingualism, multilingualism, second language acquisition, and third language acquisition.

All in all, this book is an impressive undertaking in its breadth, depth, and insight that readers will look to as a state-of-the-art source on the synthesis of theoretical and empirical studies of reflexive pronouns. Though young at its current stage, the ERA model presented in the book incorporates pragmatics and syntax to search for the antecedents of reflexives, which proposes a promising path to tackle the complex aspects of reflexive interpretation across languages. Moreover, the model also provides an innovative and unified solution to delineate reflexive interpretation across languages, informing and enlightening relevant empirical studies. As researchers, we find this book most up-to-date, well-organized, thought-provoking, and extremely useful to serve as a theoretical framework for empirical studies in areas of language acquisition, bilingualism, multilingualism, neurolinguistics, and clinical studies. Therefore, we would like to highly recommend it to anyone who is interested in the relevant research mentioned above.

## Author Contributions

LZ and HL selected the book and collaborated to prepare the draft. XZ provided valuable ideas and revised the draft. All the authors listed have made a substantial, direct and intellectual contributions to the work.

## Conflict of Interest

The authors declare that the research was conducted in the absence of any commercial or financial relationships that could be construed as a potential conflict of interest.
